# Intra-assemblage variation in the macro-blade assemblage from the 1963 excavation at Shuidonggou locality 1, northern China, in the context of regional variation

**DOI:** 10.1371/journal.pone.0234576

**Published:** 2020-06-15

**Authors:** Feng Li, Steven L. Kuhn, Fu-you Chen, Xing Gao

**Affiliations:** 1 Key Laboratory of Vertebrate Evolution and Human Origins of Chinese Academy of Sciences, Institute of Vertebrate Paleontology and Paleoanthropology, Chinese Academy of Sciences, Beijing, China; 2 CAS Center for Excellence in Life and Paleoenvironment, Beijing, China; 3 School of Anthropology, University of Arizona, Tucson, AZ, United States of America; 4 University of Chinese Academy of Sciences, Beijing, China; University at Buffalo - The State University of New York, UNITED STATES

## Abstract

The emergence of the Upper Paleolithic and regional variability in early Upper Paleolithic industries are prominent topics in Paleolithic archaeology, with special relevance to the dispersal and differentiation of early modern human cultures across Eurasia. The so-called Initial Upper Paleolithic (IUP) has been considered a key element in the emergence of the Upper Paleolithic in northern Asia. Here, we examine the intra-assemblage variation in the collection from the 1963 excavation at Shuidonggou locality 1, a major IUP site in northern China. We combine technological and quantitative attribute analyses to investigate the variety of core reduction sequences and tool manufacture behaviors at the site. A range of core reduction sequences have been documented at Shuidonggou locality 1, including both simple core reduction and prepared core reduction yielding laminar (blade-like) products. The simple core reduction component may due to mixed non-IUP assemblages from different archaeological layers. Among the laminar core reduction sequences, the main strategy involves asymmetrical exploitation of the broad face of core blank, producing blades and elongate flakes, and resembling a recurrent Levallois blade method *sensu lato*. We compare Shuidonggou laminar blank production with that of IUP assemblages in the Siberian Altai, northern Mongolia, and the Transbaikal region. The comparison demonstrates a general consistency to the basic blank production in IUP assemblages across northern Asia, with some regional variation. The results suggest a multi-directional model of diffusion of the IUP in northeast Asia.

## Introduction

Prior to the availability of genetic information, models of cultural diffusion and population dispersals, including the dispersals of modern humans into Asia, were based mainly on archaeological materials such as lithic artifacts. For many years, the southern route of modern human dispersals into East and South Asia was the main focus of attention [1–6]. However, more recently researchers have suggested an independent northern route of modern human dispersal across eastern Eurasia, based both on genetic evidence and general comparisons between lithic assemblages grouped together as Initial Upper Paleolithic (IUP) or early Upper Paleolithic [7–12]. The term IUP was generally used to describe the earliest Upper Paleolithic assemblages with Levallois elements in blade production [13–15]. Assemblages with these characteristics are distributed broadly across Eurasia, from the eastern Mediterranean to northeast Asia. In northeast Asia, IUP assemblages have been documented in Siberia, Mongolia, as well as northern China, with particular “hot spots” in the areas surrounding Baikal lake, in northern Mongolia, and in the Siberian Altai [16–21]. Our main aim here is to examine the intra-assemblage variation in the collection from the 1963 excavation at Shuidonggou locality 1 (SDG 1) in northern China, in an effort to situate the site and assemblage within the range of IUP variability in northeast Asia. We combine technological and quantitative attribute analyses to investigate the variety of core reduction sequences and tool manufacture behaviors at the site. The technological strategies of the occupants of SDG 1 are then compared with assemblages in the Siberian Altai, northern Mongolia, and the Transbaikal region to provide a more complete picture of regional variation and possible alternative scenarios of human dispersal and/or cultural diffusion.

## Materials and methods

### Ethics statement

All lithic specimens reported in this paper are housed in the Institute of Vertebrate Paleontology and Paleoanthropology, Chinese Academy of Sciences (IVPP, CAS). Access to these specimens is granted by the IVPP, CAS. Scholars who are interested in the materials presenting in the paper would be able to access these materials with the permission of the IVPP, CAS. Contact information can be found on the institute website (http://www.ivpp.cas.cn/), and scholars could also contact the IVPP through the corresponding author of this paper.

### Brief history of the excavations at SDG1

**Fig 1:**
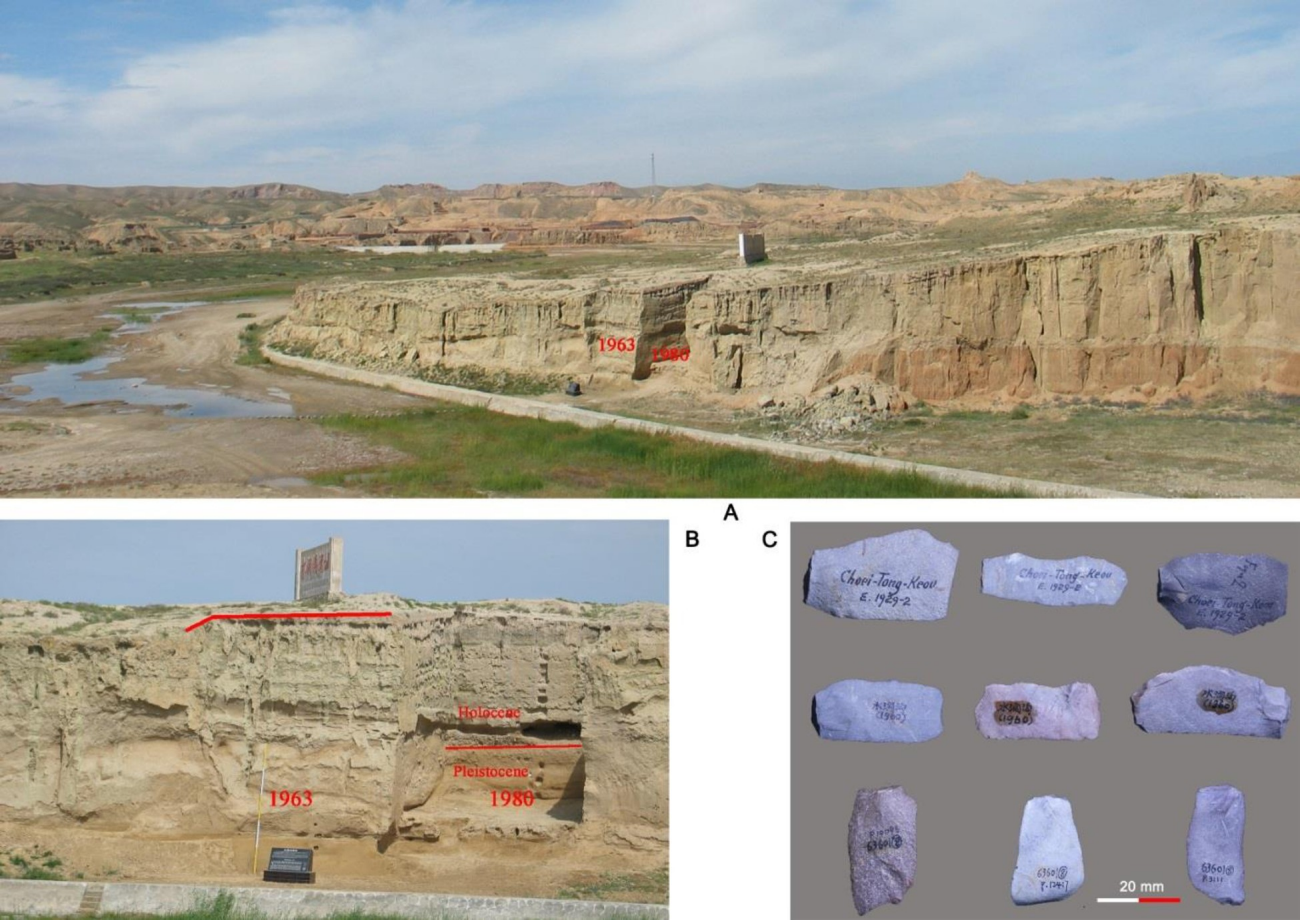
Photos of Shuidonggou locality 1 showing the profile and different excavation areas: **A**, landscape at SDG 1; **B**, a close look at the SDG 1 profile showing locations of the 1963 and 1980 trenches; **C**, A photo showing the labeling information from different excavation campaigns: top, 1920s (collected during the first excavation in 1923 or from the back dirt of the 1923 excavation later); middle, 1960; bottom, 1963.

As one of the five localities identified during the first phase of research in the Shuidonggou area, SDG 1 is among the most intensively investigated Paleolithic sites in China. Four separate excavation campaigns were conducted at the site between 1923 and 1980 (Fig 1). In total, more than 150 m^2^ were exposed at SDG1, yielding large samples of lithic artifacts, although the earlier collections are not easy to access. The locality was initially excavated in 1923 by French scholars Émile Licent and Pierre Teilhard de Chardin [22, 23]. Although detailed records are not available, the first excavations were conducted over an area of more than 80 m^2^. Large quantities of archaeological materials were exposed and removed or collected, including faunal remains, hearths, and roughly 300 kilos of lithic artifacts [24]. The lithic material from this excavation was studied in detail by Henry Breuil [23]. Most of this collection is currently stored in the Muséum National D’Histoire Naturelle in Paris, France. A very small number of pieces are stored in the IVPP, CAS. The second phase of excavations at SDG 1 took place in 1960, when a Sino-Soviet team returned to the site to excavate a somewhat smaller area (ca. 36 m^2^) [24]. Around 2000 stone artifacts were collected. Unfortunately most of the materials from this campaign may have been lost, except for around 300 pieces stored in the IVPP, CAS. Because of the imprecise digging methods used in the 1960 excavations many artifacts were missed: during the 1963 excavation campaign, IVPP researchers recovered a large sample of artifacts from the back dirt of the 1960 excavations. A report on the stratigraphy and a small number of typical retouched tools was published by Jia et al. in Chinese [25]. Two subsequent campaigns were conducted by Chinese teams at SDG 1, the first in 1963 and the second in 1980. This paper examines the 1963 collection, and the excavation will be described in detail in the next section. The 1980 excavation was organized by Ningxia Museum and the Survey Team of Regional Geology Bureau of Geology of Ningxia Hui Autonomous Region. An area of 52 m^2^ were exposed and 6700 stone artifacts, 63 bone fragments, and several ash features were discovered [24, 26]. This collection is currently housed in the Provincial Institute for Archaeology in Ningxia Hui Autonomous Region in Yinchuan City. The lithic materials from the 1980 excavations have been studied by several different research groups, and a monograph and many research articles have been published [20, 24, 27, 28].

### The SDG 1963 excavation

While SDG1 was originally considered to be exclusively a Paleolithic locality, the 1963 excavation, led by W.C. Pei, also identified a Neolithic component in the upper layers [29]. Due to the loss of some field documents from this campaign, the size of the excavated area is currently uncertain. The collection is currently housed in the IVPP, CAS. The collection includes specimens ranging from large cores to small chips less than 20 mm in length. Therefore, we are fairly certain that excavators employed sieves, at least partially, in the 1963 excavation, although we do not know what mesh sizes they used. Responsibility for the study of the 1963 assemblage was initially divided among two research groups of the IVPP, one focusing on core reduction and blank production, and the other focusing on retouched tools. The sample we present here includes both groups of artifacts, which represent the more or less complete assemblage from the Paleolithic horizon of the 1963 excavation.

Although the stratigraphic section of the 1963 excavation has never been published, we can determine the layer provenance of each artifact based on the layer number written on the specimen (Fig 1C). Artifacts from Layer 8, attributed to the main Paleolithic horizon, are the main concern of this paper. The collection used in our study includes 5476 specimens, which makes it the largest intact collection from the Paleolithic layers of the SDG 1 site.

### Stratigraphy and dating of the site

Although details are missing, the stratigraphic section of the 1963 excavation has been divided into 8 layers. Layer 8, the main Pleistocene cultural deposit, has a total thickness of at least 2 m. It was formed by the accumulation of the second terrace of a small branch of the Yellow River. This layer consists mainly of gray-yellow loess-like fine sand, though through the long history of the research of this site various publications have described the section differently. In the latest publication, Liu et al. [30] describe four sub-strata within ‘Layer 8’ including: grayish yellow silt, blocky structure, calcareous cement with some nodules; grayish yellow silt, blocky structure, a few redoximorphic mottles; grayish yellow fine sand, coarse sand, planar bedding; and light grayish yellow silt, planar bedding, redoximorphic mottles.

Due to the loss of field documents and the coarse excavation method, the stratigraphic associations between the archaeological materials and dated samples from SDG 1 are poorly understood and the age of the lithic assemblages remains uncertain [21, 31]. The best current estimates for the age of Paleolithic layers at SDG 1, based on a combination of dates from this site and from its sister locality SDG 2, are between 34 ka and 41 ka (calibrated ages) [31, 32], but it is possible that the first occupation occurred as early as 41‒46 ka [33, 34]. Ongoing excavations at SDG 1 should resolve the uncertainties about the depositional history of the site and the age of the earliest assemblages in the near future.

### Methods of lithic analysis

The aim of this study is to fully document the available lithic artifacts from Paleolithic layers of the 1963 excavation at SDG 1 and to investigate potential variation within the Paleolithic assemblage. The large collection from 1963 has only been mentioned briefly in some review publications [35]. In the sections that follow we combine several different, complementary approaches to lithic analysis.

The study centers on an evaluation of the methods of core reduction that knappers performed at the site. This part of the study uses a series of observations on cores, blanks, and the “technological pieces” (crested blades, *eclats debordants*, etc.) to reconstruct the basic methods of core reduction. Core forms at SDG 1 are described in terms of the organization of the platforms and flaking surfaces, the angles and treatment of the platforms, and the main products. Categories of core forms used here include Levallois-centripetal, Levallois-unidirectional/bidirectional, edge-faceted, prismatic/subprismatic, burin core, discoid, and polyhedral. Levallois-unidirectional/bidirectional cores are the most common forms in the SDG 1 assemblages and the other IUP assemblages in northeast Asia: they have also been called Levallois-like blade cores [14, 31] or flat-faced cores [27] in the literature. The frequency of different reduction methods is quantified in terms of major core forms. “Technological pieces” provide additional information about core shaping and maintenance that may not be preserved on individual cores.

Metrical and attribute data inform on technological choices by providing quantitative information about the features of individual artifact. We apply this approach mainly to the blanks and retouched pieces to provide supplementary information to the results of the analysis of cores, and to explore preferences for certain blank morphologies. Only stone artifacts ≥ 30 mm in maximum dimension were included in the sample for full metric and attribute analysis. A reduced set of observations were made of small flakes, chips and fragments.

The attribute analysis follows the list made by Scerri et al. [36] with some alteration: we have added attributes representing platform maintenance, dorsal scar pattern, and general morphology of the blanks. Small artifacts (< 30mm) were sorted by raw material as an aid in evaluating the level of on-site production. Although the 1963 excavation did not apply the same excavation and recovery methods we typically use today, the sample of small pieces still provides some meaningful information about site formation and segmentation of knapping at SDG 1.

Typological classification of tool types facilitates comparisons with other assemblages and provides information about selection of blank types for transformation into certain forms. Because both Middle and Upper Paleolithic artifact types are abundant in the collection, we follow the commonly used typological lists of both F. Bordes [37, 38], and D. de Sonneville-Bordes and J. Perrot [39–43], both of which have been applied to the Shuidonggou assemblages in previous studies [27].

## Results

### Procurement and use of raw materials

Several different raw materials were used to produce stone artifacts at SDG 1. We grouped the raw materials into four broad categories according to lithology. Some of these categories may include multiple rock types according to strict petrological identification. However, the nature of cortex, when preserved, suggests that most raw materials were collected as pebbles from nearby fluvial deposits. Because the rocks came from secondary sources, fine-grained distinctions among rock types are not important in this case. Based upon two independent raw material surveys [44, 45], gravel deposits in the Shuidonggou area are dominated by siliceous dolomite and quartzite, followed by chert and sandstone. Clasts in these deposits range from sub-centimeter pebbles to cobbles as large as 20–30 cm in length. The siliceous dolomite (some also define it as siliceous limestone [45]) in the Shuidonggou area is generally fine-grained and relatively easy to flake, although it sometimes includes beddings of chert resulting in irregular fracture. Quartzite in the gravel deposits is relatively homogeneous though coarse-grained. The local chert pebbles typically contain many flaws and incipient fractures.

In order from most to least abundant, the main raw materials exploited by knappers at SDG 1 include silicified dolomite (61.4%), quartzite (26.3%), chert (12.2%), and quartz and others (0.1%) (Table 1). The overall frequency of the first two main raw material types is consistent with what Peng et al. [20] observed in the 1980 collection (68.4% silicified dolomite and 22.3% quartzite).

**Table 1:** Quantitative description of the SDG 1963 assemblage divided by raw material in Layer 8.

Raw material	Core	Blank (≥30 mm)	Retouched pieces	Debris (<30 mm)	Total
Silicified dolomite	176 (5.2%)	1380 (41.1%)	286 (8.5%)	1520 (45.2%)	**3362 (61.4%)**
Quartzite	53 (3.7%)	641 (44.6%)	114 (7.9%)	630 (43.8%)	**1438 (26.3%)**
Chert	20 (3%)	110 (16.5%)	121 (18.2%)	415 (62.3%)	**666 (12.2%)**
Quartz and others		5 (50%)	3 (30%)	2 (20%)	**10 (0.1%)**
Total	**249 (4.5%)**	**2136 (39%)**	**524 (9.6%)**	**2567 (46.9%)**	**5476 (100%)**

**Fig 2:**
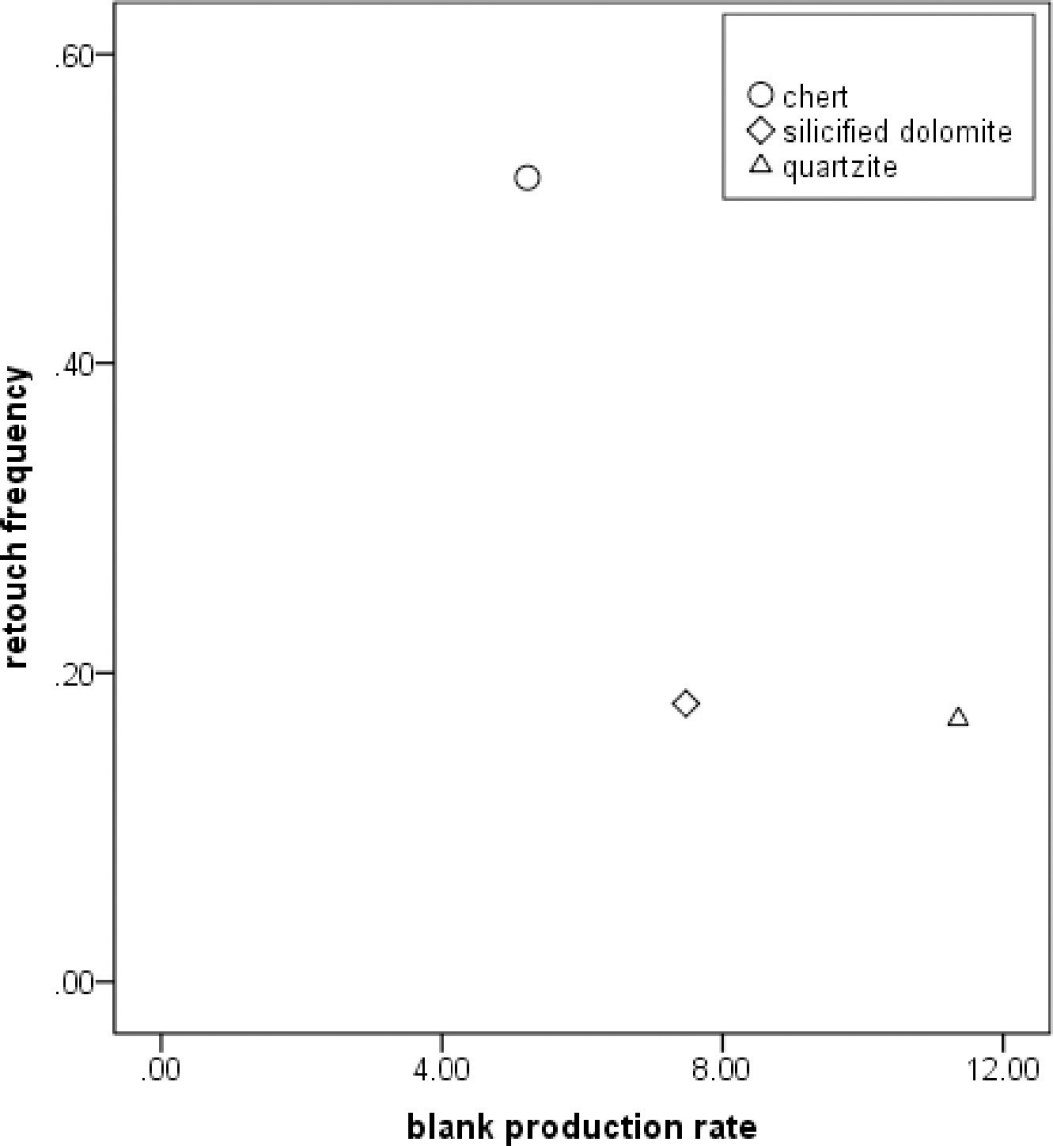
Raw material exploitation of the SDG 1963 assemblage from Layer 8.

The frequencies of cores, blanks, retouched pieces and debris are quite similar for the two main raw materials (Table 1). However, the retouch frequency for chert blanks (53%) is much higher than for quartzite (16%) and silicified dolomite (18%) blanks (Fig 2). One possibility is that chert was preferred over quartzite and silicified dolomite for making certain kinds of implements. It could also be the case that blanks of other raw materials were simply more suitable for use without retouch than chert blanks. The higher frequency (62.3%) of small fragments within the chert sample is probably due to the nature of the cherts used at SDG 1, which are both brittle and full of internal flaws. The effects of this can be observed on the blank production rate, in that the ratio of large (≥30mm) blanks per core for chert is the lowest of the three main raw material types (Fig 2).

### Analysis of debitage

**Fig 3:**
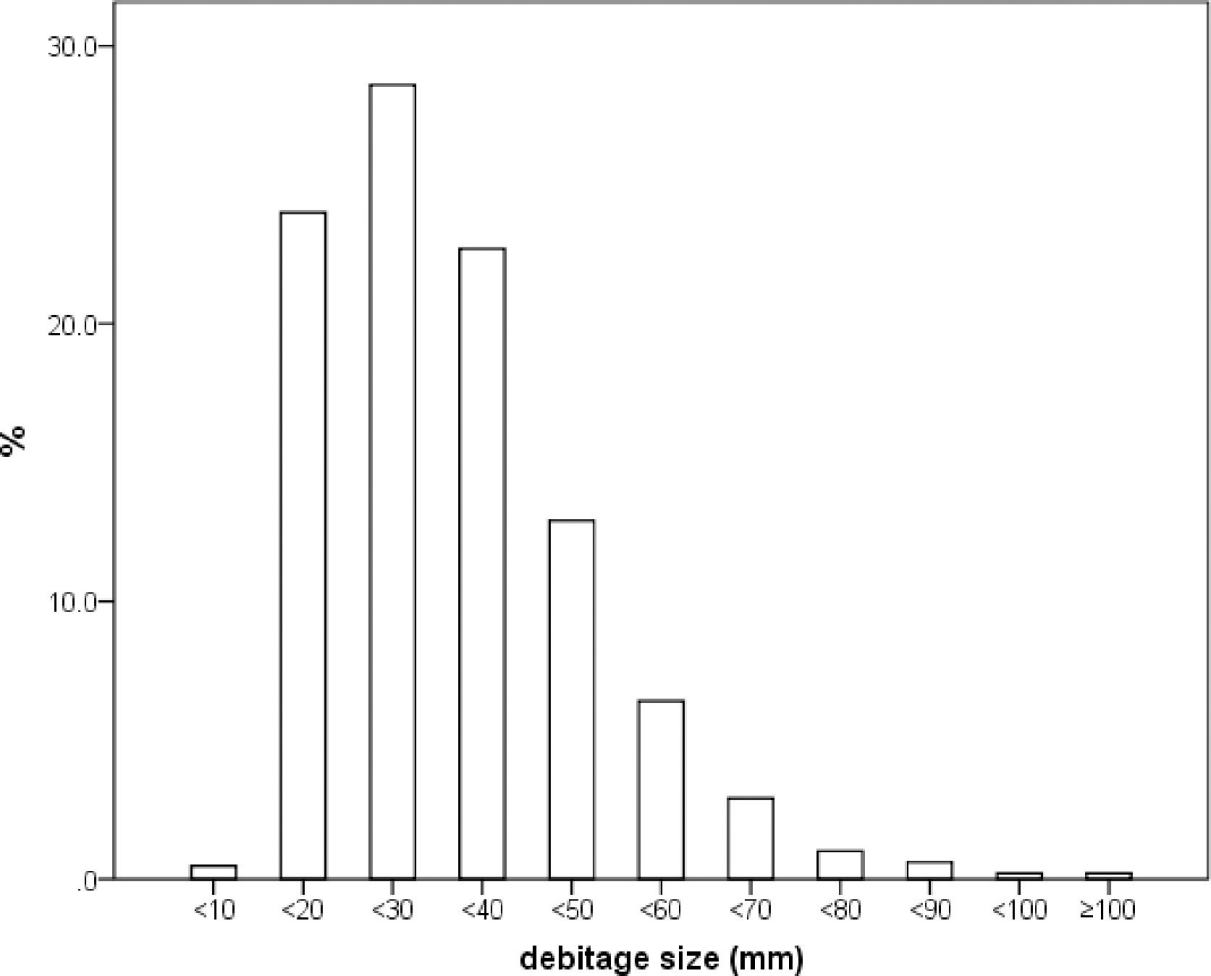
Debitage size distribution (mm) of the SDG 1963 assemblage from Layer 8.

We are not sure of the size of the sieves used in the 1963 excavation. However, we can be confident that at least 10 mm mesh was used, based on the high frequency of small debitage < 20 mm in maximum dimension (n = 1252, 24%) and the near absence of specimens < 10 mm (n = 24, 0.5%) (Fig 3). Although no experimental data are available for comparable raw materials and reduction practices found at SDG 1, the size profile of the material from the 1963 excavation shows a similar frequency of fragments from 10 mm to 30 mm (52.6%) to the experimental data reported by Schick (56.8%) (Fig 3) [46]. This suggests that the 1963 assemblage from Layer 8 is relatively complete, and that colluvial or alluvial forces have not removed a large part of the small material. Because of the absence of contextual information from the excavation we cannot evaluate the site formation in any detail. However, the presence of the full array of reduction products and byproducts, and the relatively high frequency of small material, suggest that much knapping took place at the site, and that the assemblage should provide information about complete *chaînes opératoire*.

### Core forms and reduction strategies

The methods of core reduction were an integral part of the technological system of IUP knappers. We chose to use a taxonomy of cores frequently used by other researchers working in northeast Asia (Table 2) to classify the large sample of cores from the 1963 excavations at SDG 1. We do not provide precise metric data in this study. Instead, following other studies, we focused on the presence and frequencies of cores likely to represent different reduction procedures. Table 2 summarizes the core forms found in the 1963 assemblage from SDG 1. Two broad groups of cores can be identified. Once consists of simple cores without systematic preparation of platforms and flaking surfaces, and the other of more organized cores with deliberate preparation of platforms and shaping of flaking surfaces. The simple cores almost always yielded flakes. Simple unipolar cores (n = 21, 8.4%) are the most abundant in this group: other types presenting in low frequency include tested pieces, chopper/chopping tools, polyhedrons, and bipolar cores. Among the organized core types, Levallois-unidirectional/bidirectional (n = 94, 37.8%), prismatic/subprismatic (n = 35, 14.1%), and edge-faceted (n = 13, 5.2%) cores were used mainly for production of blades and laminar pieces. Five of the specimens in the group of organized cores (2%), with highly variable morphology, can be classified as bladelet cores based on the sizes of the final detachments. Among them are two burin cores. Levallois-centripetal (n = 6, 2.4%) and discoidal (n = 3, 1.2%) cores are also present but rare overall.

**Table 2:** Count and frequency of core forms in the SDG 1963 assemblage from Layer 8.

Core type	Numbers	Percentage
Tested	13	5.2%
Chopper/chopping tool	1	0.4%
Core preform	11	4.4%
Simple unipolar	21	8.4%
Polyhedron	6	2.4%
Levallois_Centripetal	6	2.4%
Levallois_Uni/Bidirectional	94	37.8%
Edge-faced	13	5.2%
Prismatic/Subprismatic	35	14.1%
Prismatic-Levallois_Unidir	10	4%
Burin core	2	0.8%
Bladelet core	3	1.2%
Discoid	3	1.2%
Fragment	31	12.5%
Total	249	100%

**Fig 4:**
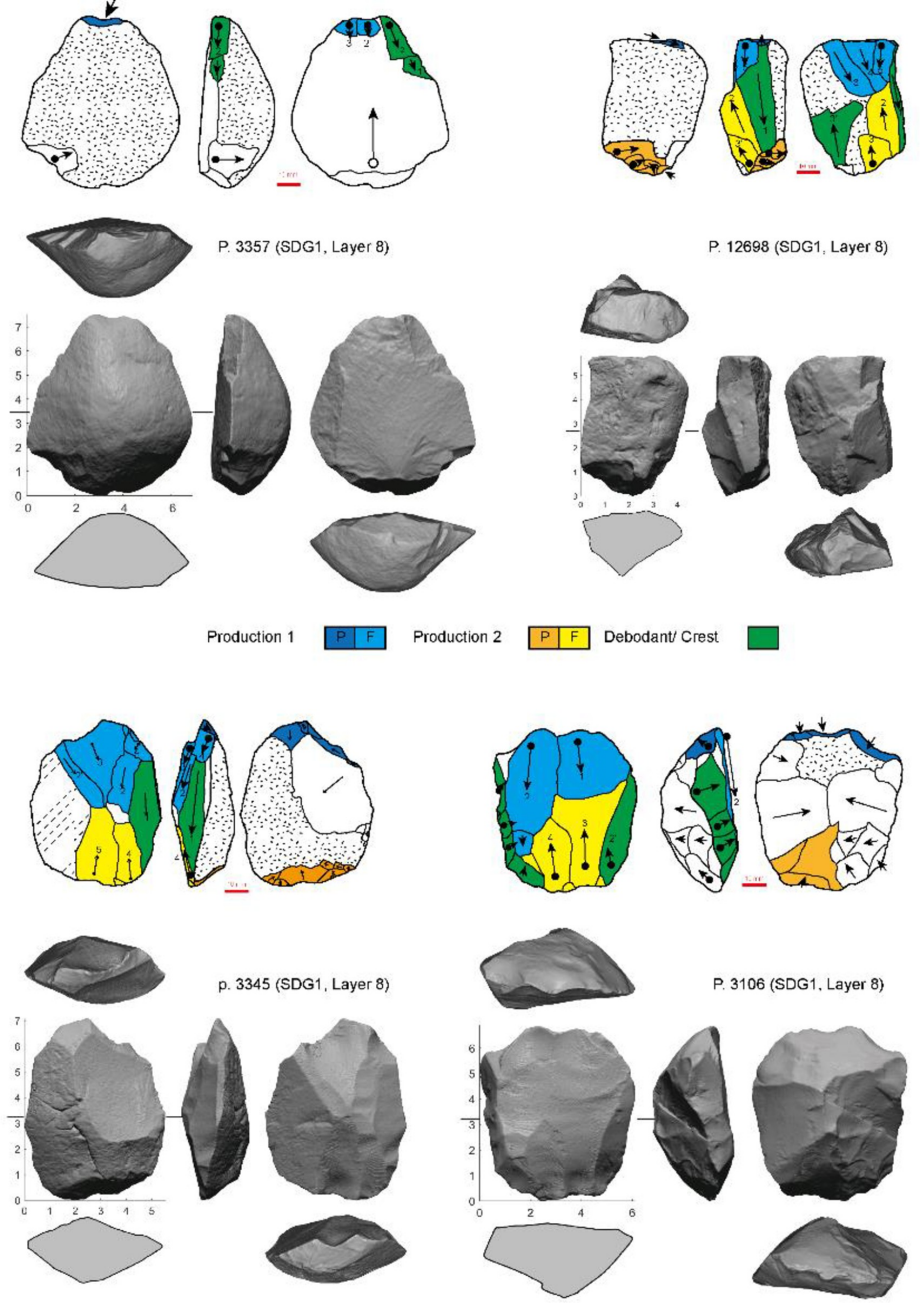
Examples of Levallois-unidirectional/bidirectional cores showing the initial stage and maintenance (different series of production are indicated by colors. P: Platform; F: Flaking surface).

**Fig 5:**
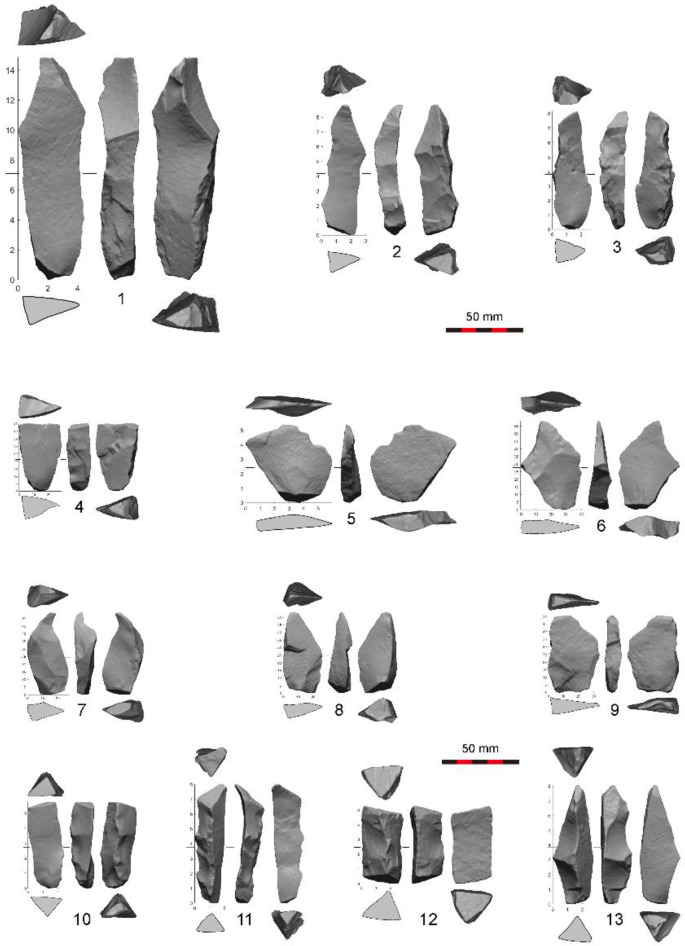
Examples of debordant crests (1–4) and flakes (5–9) and central crests (10–13) in the assemblage from Layer 8, SDG 1963.

**Fig 6:**
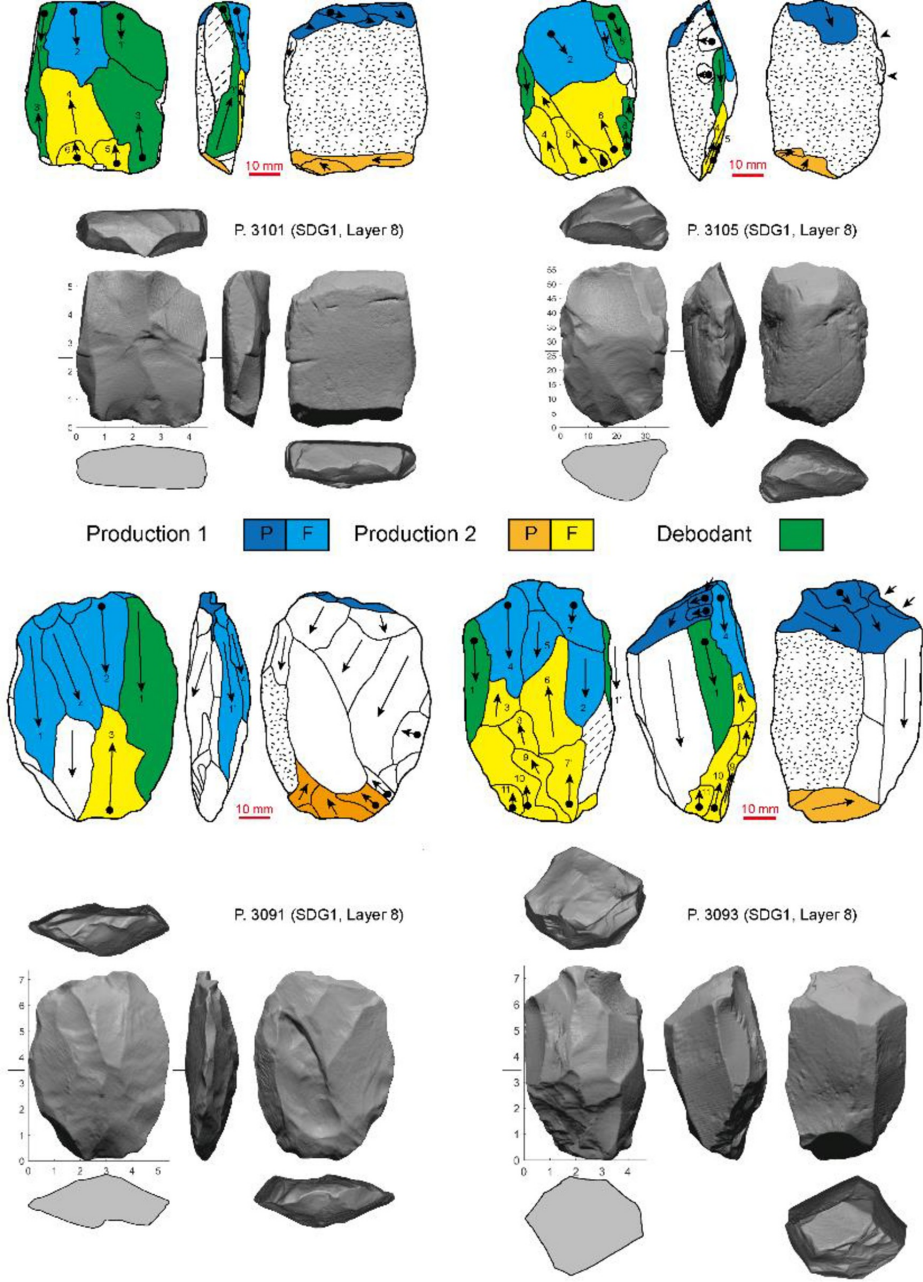
Examples of various Levallois-unidirectional/bidirectional cores in the SDG 1963 assemblage from Layer 8 (different series of production are indicated by colors. P: Platform; F: Flaking surface).

The Levallois-unidirectional/bidirectional cores at SDG 1 mostly resemble byproducts of a recurrent Levallois system *sensu lato* of reduction, although some of the flaking surfaces are not precisely hierarchically arranged [28]. Typically, knappers prepared one or two opposed platforms, and detached elongated flakes and blades from one relatively flat and broad surface of the core, often bidirectionally. Several preforms or incompletely prepared cores demonstrate that these cores were made on either flat pebbles or thick flakes (or occasionally rectilinear chunks) (Fig 4). The platform was prepared by flaking or faceting, and often orientated oblique to the longitudinal axis of the piece. The transverse convexity of the Levallois surface was established and maintained either by striking *debordant* flakes along one edge (sometimes two edges) or by lateral shaping, resulting in a lateral crested blade. Often both techniques were used on the same core. Longitudinal convexity was maintained either by shaping the distal end, or by the negative scars from opposed removals (in the case of cores with two opposed striking platforms). *Debordant* flakes and blades (n = 248) and crests (n = 37) are both present in the assemblage, with the former being much more common (Table 3 and Fig 5). The backs of the cores are generally flat or gently convex, covered by cortex or invasive flake scars. The overall morphology of Levallois-unidirectional/bidirectional cores vary due to the initial shape of core forms and the extent of reduction, resulting in a variety of forms, with very flat to more convex flaking faces (Fig 6). These cores produced large rectangular or convergent flakes and blades, often with faceted platforms, flat longitudinal profiles, parallel dorsal scars, and exterior platform angles (EPA) around 80°.

**Table 3:** Frequency of complete blanks^a^ divided by platform types in the SDG 1963 assemblage from Layer 8.

Platform types	Generalized Flakes	Laminar blanks (blades and bladelets)	Levallois flakes	Levallois points	Core tablets	Debordants	Crests	Total
Cortical	160 (14.3%)	12 (3%)			3 (15.8%)	20 (8.1%)	7 (18.9%)	202 (10.8%)
Cortical and Plain	15 (1.3%)	12 (3%)				6 (2.4%)	2 (5.4%)	35 (1.9%)
Plain	598 (53.6%)	211 (52%)	1 (2.9%)		7 (36.8%)	127 (51.2%)	17 (46%)	961 (51.6%)
Dihedral	82 (7.4%)	21 (5.2%)	2 (6%)		4 (21.1%)	16 (6.5%)	1 (2.7)	126 (6.8%)
Facetted	140 (12.6%)	122 (30%)	25 (73.5%)	3 (75%)	4 (21.1%)	73 (29.4%)	10 (27%)	377 (20.2%)
*En chapeau de gendarme*			5 (14.7%)	1 (25%)				6 (0.3%)
Crushed	105 (9.4%)	22 (5.4%)				6 (2.4%)		133 (7.1%)
Not applicable	16 (1.4%)	6 (1.4%)	1 (2.9%)		1 (5.2%)			24 (1.3%)
**Total**	1116 (59.9%)	406 (21.8%)	34 (1.8%)	4 (0.2%)	19 (1%)	248 (13.3%)	37 (2%)	1864

^a^ Complete blanks include whole and nearly whole (proximal and medial) flakes; retouched pieces on whole and nearly whole flakes are counted here.

**Fig 7:**
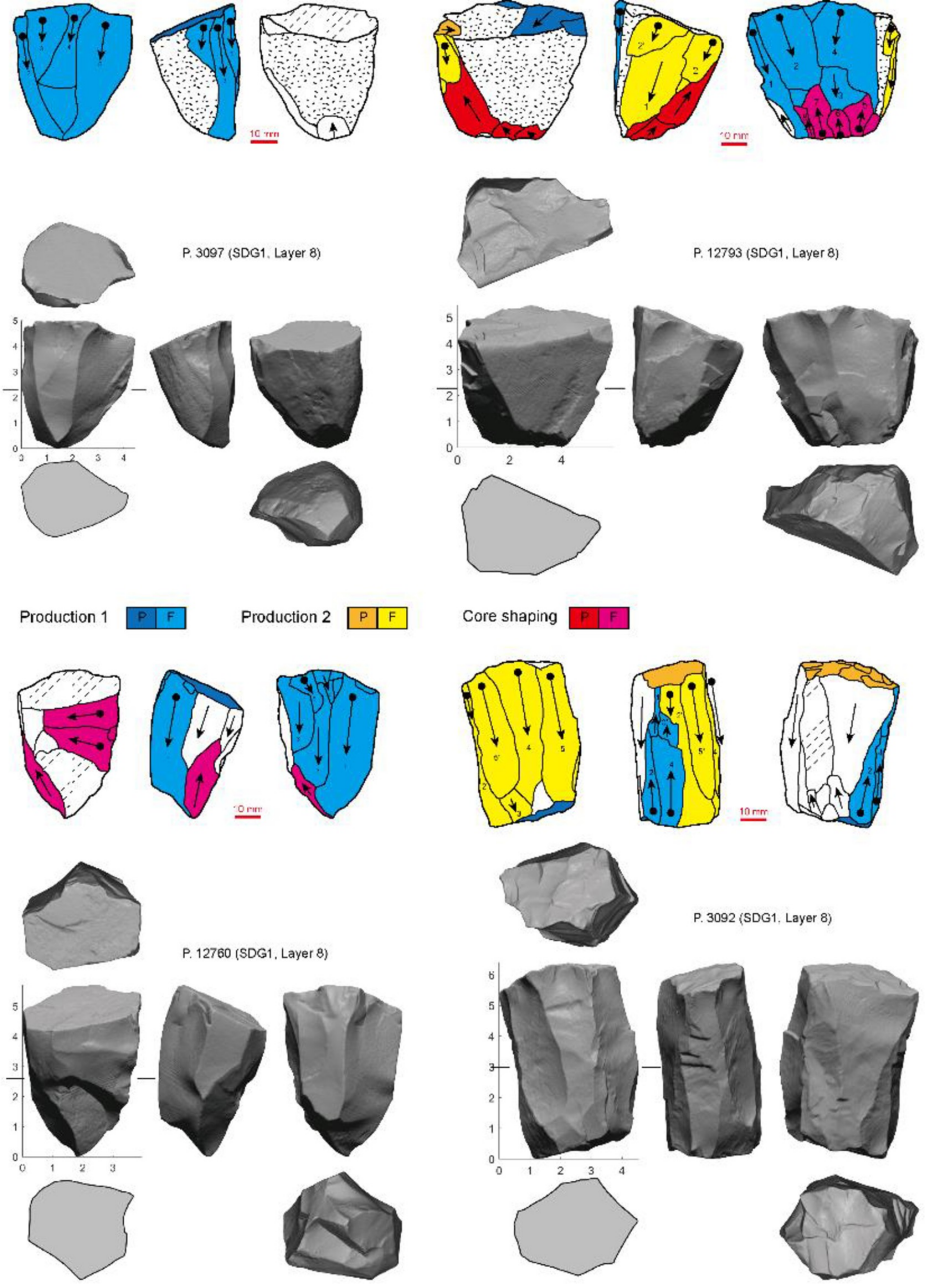
Examples of prismatic/subprismatic cores in the SDG 1963 assemblage from Layer 8 (different series of production are indicated by colors, P: Platform; F: Flaking surface).

**Fig 8:**
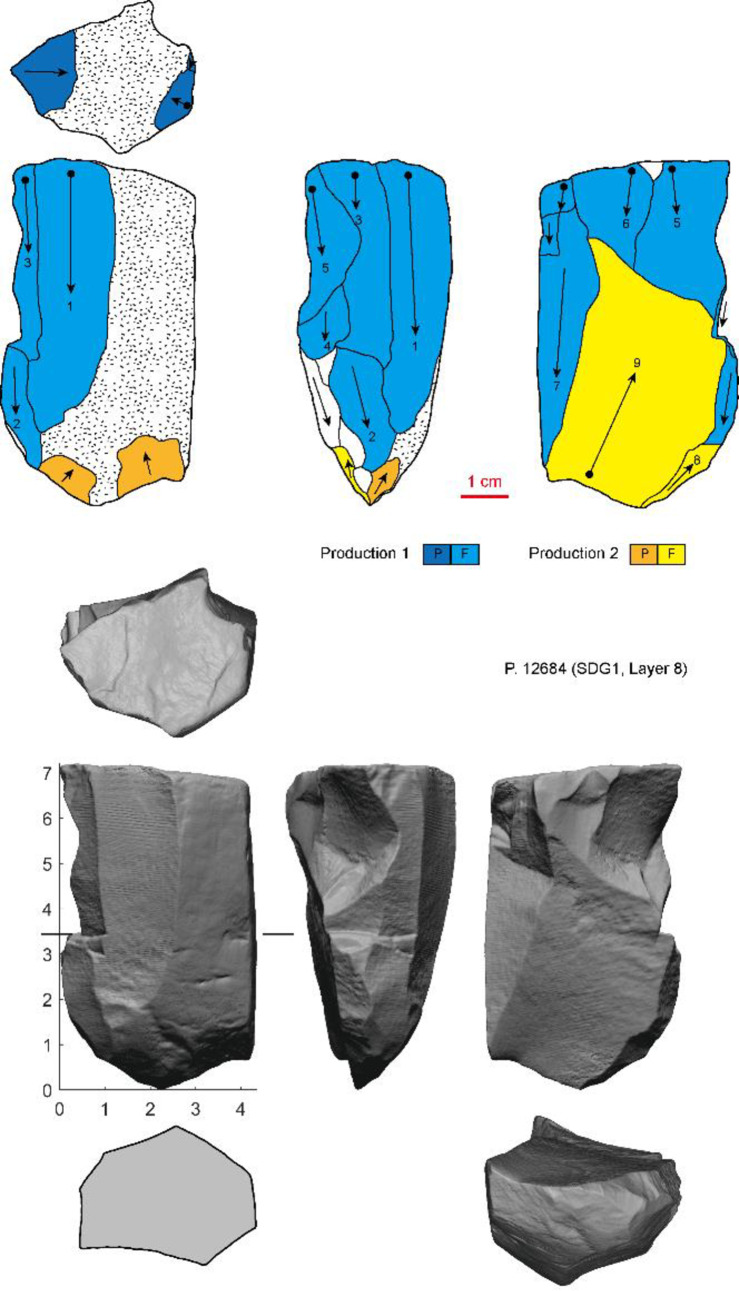
Examples of cores combing both Levallois-unidirectional/bidirectional and prismatic/subprismatic exploitation (different series of production are indicated by colors. P: Platform; F: Flaking surface).

A second group of artifacts is classified as prismatic/subprismatic cores, but two sets of procedures can be reconstructed from these cores. The first can be treated as a variation of the Levallois-unidirectional/bidirectional reduction system, often with two opposed facetted platforms and bidirectional blank removal. However, instead of maintaining a broadly convex face of detachment, knappers exploited the lateral edges of the flaking surface so that the cores were reduced more volumetrically. To test whether these cores and the typical Levallois-unidirectional/bidirectional cores are independent reduction strategies or the result of the morphological change through reduction following a single strategy, we will analyze 3D models of the cores in another paper. The second procedure shows a much clearer volumetric system, although the number of cores is small (n = 4) (Fig 7). One flat platform perpendicular to the longitudinal axis of the core was used to remove blades and elongate flakes from much of the core’s circumference, producing a conical shape. A few cores combine both systems of reduction (Fig 8). These specimens preserve two opposed platforms, one oblique and one perpendicular to the longitudinal axis. Blades and elongate flakes were detached from several sides of the perpendicular platform, which creating a circular cross-section. Flakes were produced from the opposed oblique platform using only one face of the core, resulting in a much flatter cross-section.

Edge-faceted cores, exploited along one edge of the blank, appear to represent a third set of procedures. The organization of the platforms is similar as the Levallois-unidirectional/bidirectional system, but the platforms are not faceted.

**Fig 9:**
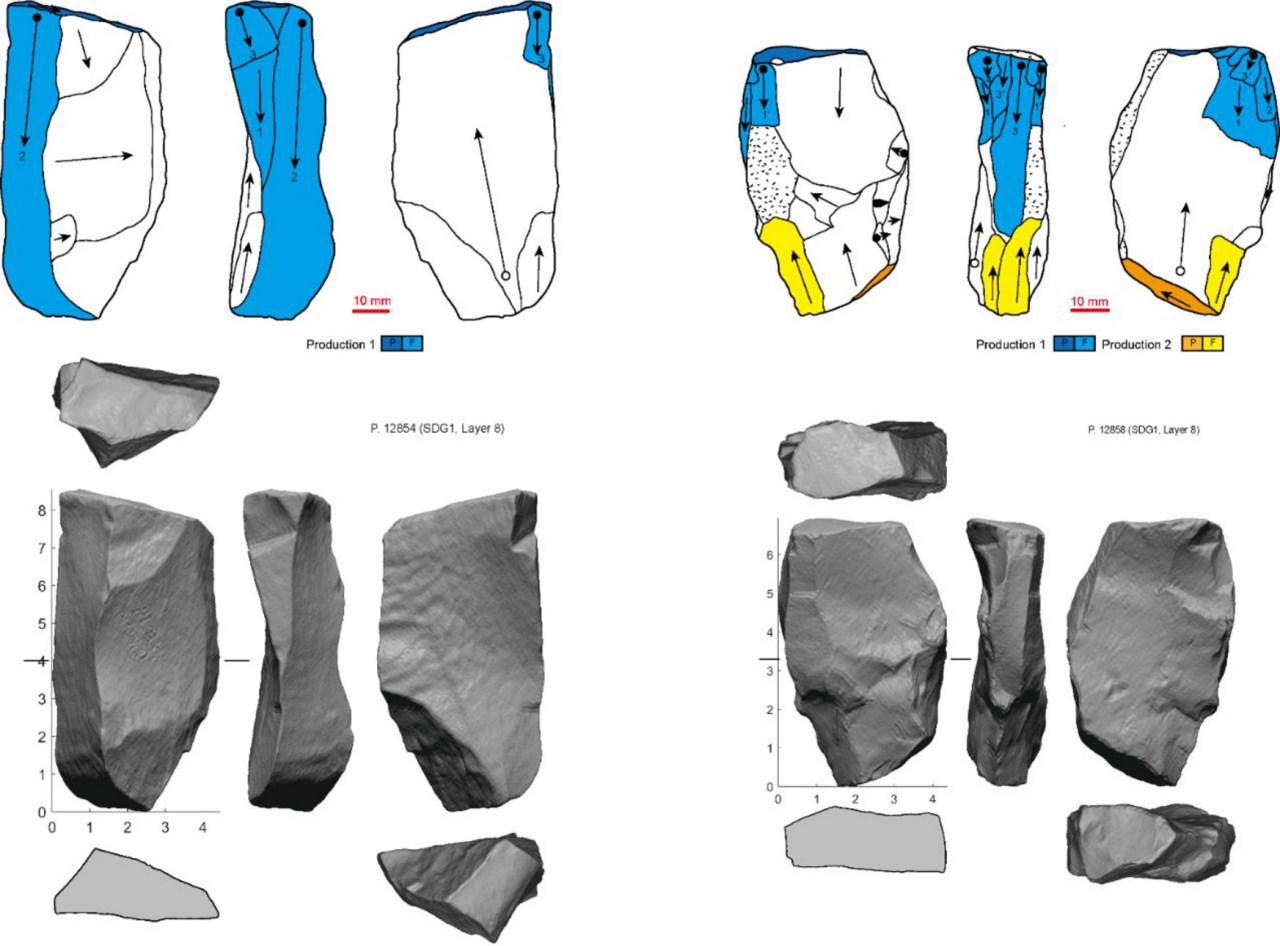
Examples of burin cores in the SDG 1963 assemblage from Layer 8 (different series of production are indicated by colors. P: Platform; F: Flaking surface).

Cores (n = 5) yielding bladelet-sized blanks (width< 12 mm) were exploited using several different systems. Two “burin cores” were made on thick elongate flakes (Fig 9). Platforms were flaked or facetted and narrow blades and bladelets were removed from one edge of the thick blanks. The other cores yielding bladelets resemble larger core forms, raising the possibility that they are simply reduced versions of larger blade cores.

### Morphology of blanks

**Fig 10:**
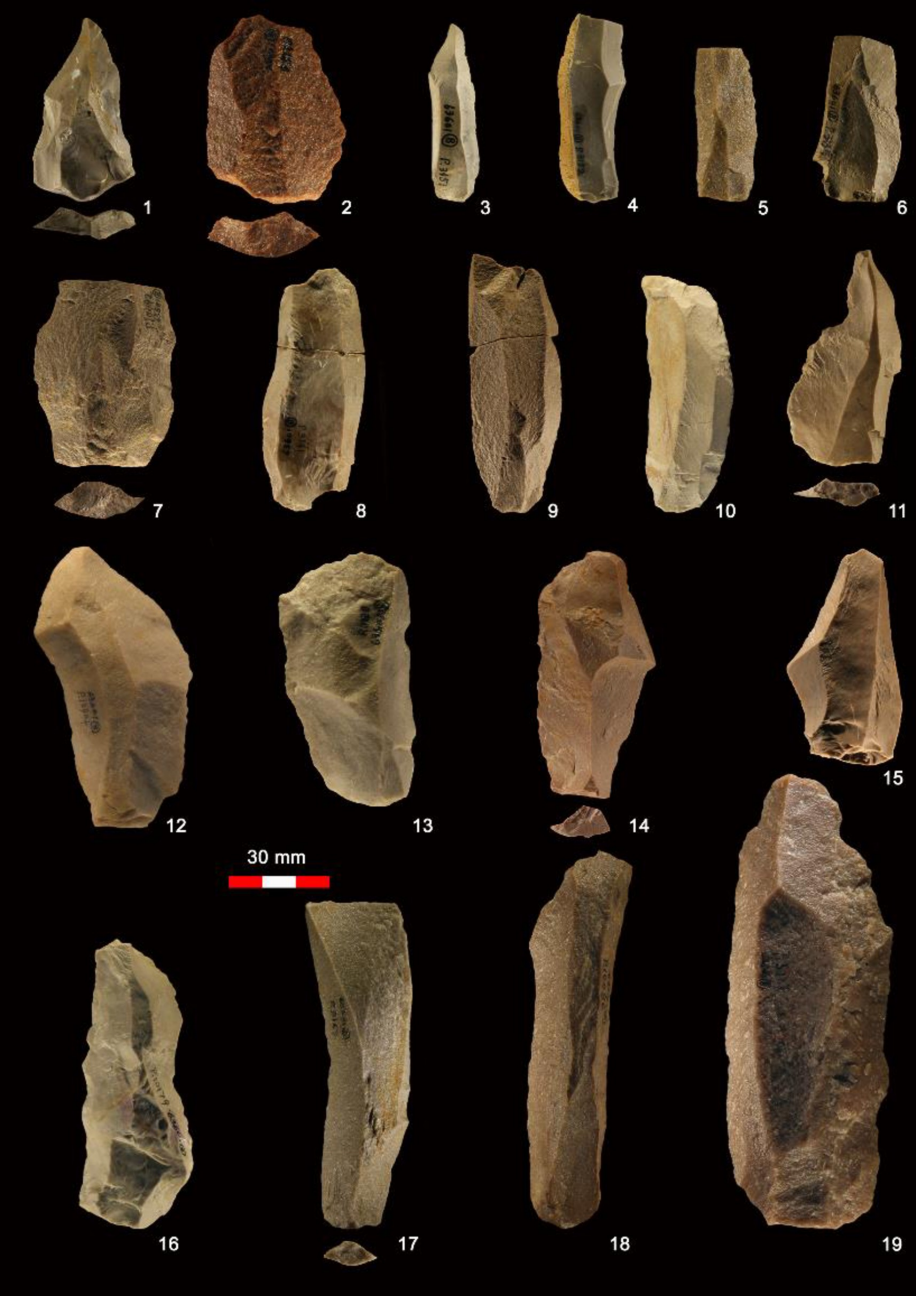
Examples of various blanks in the SDG 1963 assemblage from Layer 8: 1, Levallois point; 2, Levallois flake; 3–10, 16–19, blade; 11,15, convergent flakes; 12–14, elongated flakes.

**Fig 11:**
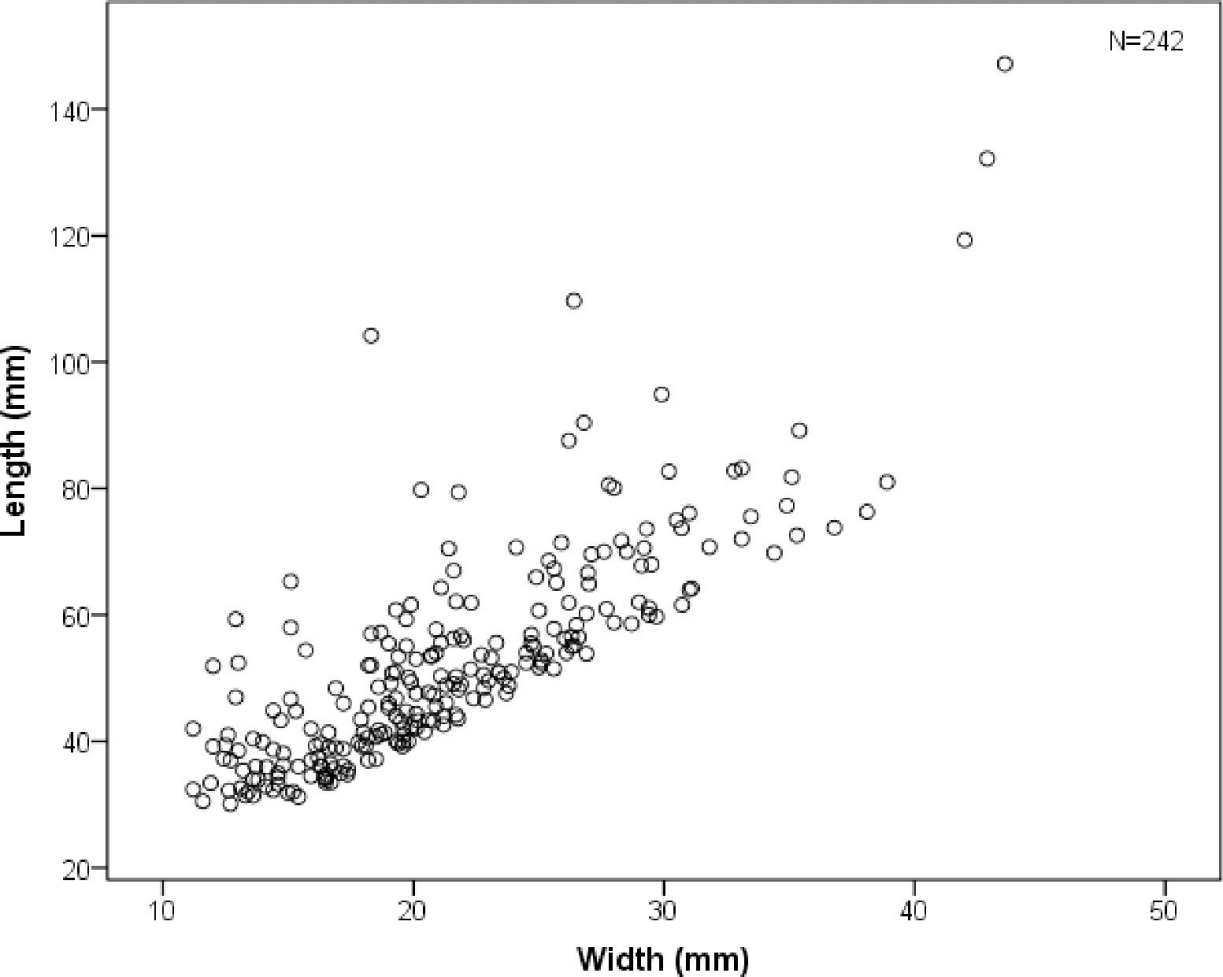
Scatter plot of blade widths and lengths (including technical pieces and nearly whole blanks with a length and width ratio larger than two) in the SDG 1963 assemblage from Layer 8.

**Fig 12:**
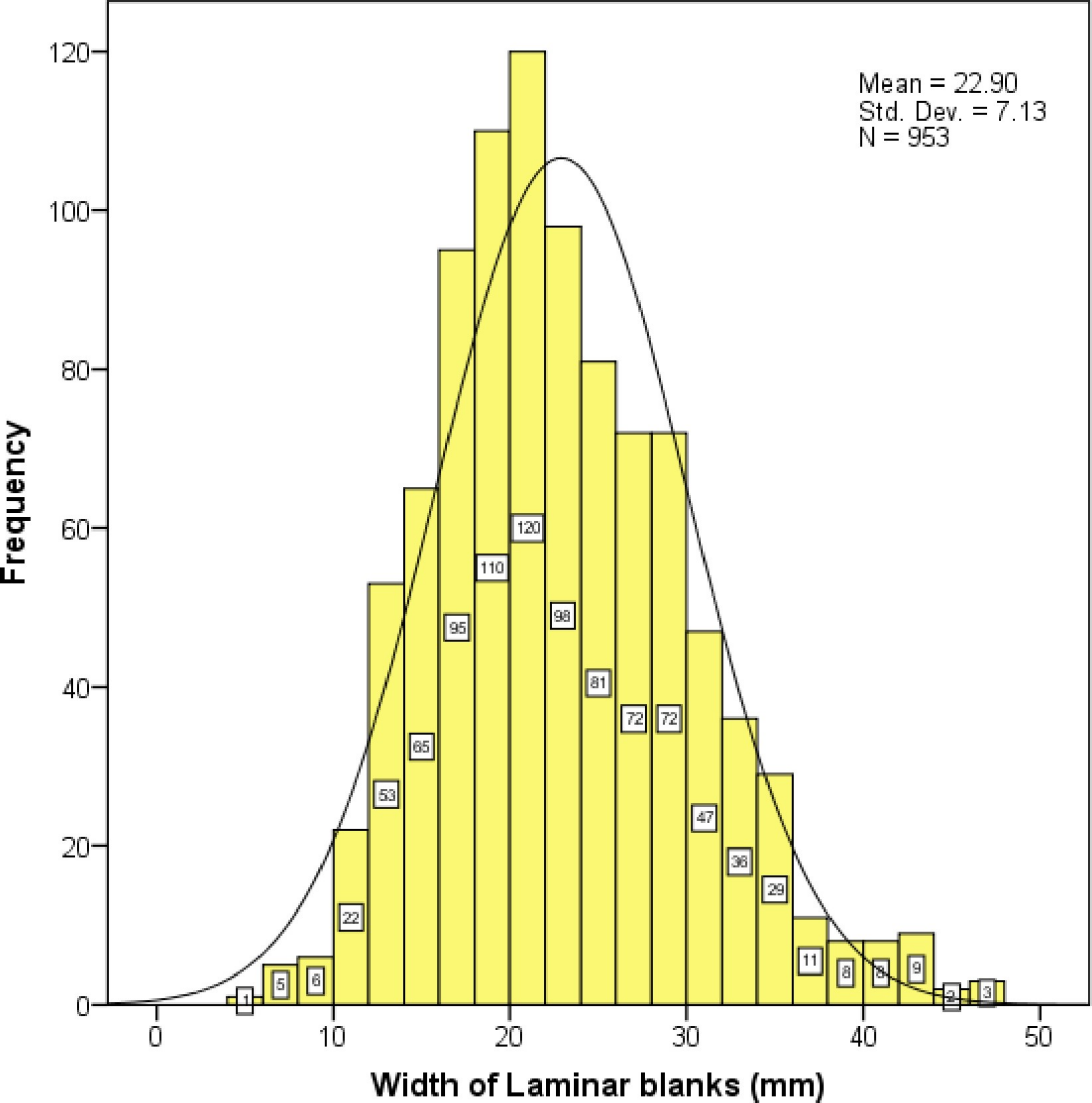
Histogram of laminar blank width (including blanks smaller than 30 mm) in the SDG 1963 assemblage from Layer 8.

The SDG 1 assemblage contains a variety of blanks (Table 3) including generalized flakes, blades, technological pieces, and pointed flakes (such as Levallois points), as documented from the blanks themselves and the negative scars left on cores (Fig 10). Generalized flakes of various morphologies and sizes make up the majority of blanks of the 1963 collection, consistent with observations on the 1980 assemblage. The frequency of morphological blades, with length > 2x (width), is relatively low (n = 180, 9.7%) among the specimens ≥ 30 mm long. However, the fragmentation rate of laminar blanks (79.7%) is quite high and many fragments with parallel sides and parallel dorsal scars can be classified as laminar blanks [47], even if the preserved dimensions do not qualify them as blades in the strict sense. In total, laminar blanks make up 21.8% of the blank assemblages (Fig 11). Some laminar specimens with a width < 12 mm can be considered bladelets. There may also be bladelet fragments in the debris category (<30 mm) which were not recorded in detail. To more fully evaluate the production of bladelets at SDG 1, we included all the laminar blanks including the small fragments (n = 285) under the 30 mm size cut-off. The widths of laminar blanks exhibit a unimodal distribution with a peak around 22 mm (Fig 12). The distribution is generally symmetrical but slightly left skewed. Given the absence of multi-modality in the width distribution it is likely that the distinction between blades and bladelets in the SDG 1 assemblage is arbitrary, and that most bladelet-sized pieces were detached from large blade cores as byproducts or as the final detachments. On one hand, the presence of a small number of burin cores and the continued use of reduced cores, indicate that the early inhabitants of SDG 1 were sometimes interested in producing very small blades. On the other hand, we note that small and large blades were transformed into similar kinds of shaped tools: there is no systematic production of backed pieces on small blanks, for example.

Based upon the observations of platform types, platform thickness, and EPA, flakes and laminar blanks tended to be produced by different methods (Table 4). Cortical and plain platforms are more often present on flakes, while facetted platforms are more abundant among laminar blanks. Many generalized flakes were probably produced during core shaping operations, such as creating the platforms on pebbles, so the higher cortical platform frequency is expected. Many platforms were chipped on the exterior edge to adjust the flaking angle, and there is no significant difference between flakes and laminar blanks in this regard. This is not surprising because many of the Levallois-unidirectional/bidirectional core surfaces yielded both flakes and laminar blanks. Grinding of the platform edge is extremely rare (n = 2). The mean platform thickness of laminar blanks is smaller, with a smaller standard deviation (mean = 6.6 mm, SD = 2.9), than platform thickness of flakes (mean = 7.5 mm, SD = 3.8).

**Table 4:** Comparison between blades and generalized flakes in the SDG 1963 assemblage from Layer 8.

Selected categories	Generalized flake	Blades	Statistical significance
Fragmentation rate	38.2%, *n* = 1548	79.7%, *n* = 706	
Whole	957	143	Chi-square = 333.63, df = 1, p<0.001
Fragment	591	563	
Platform type	*n* = 1116	*n* = 406	Chi-square = 98.432, df = 5, p<0.001
Cortical	160 (14.3%)	12 (3%)	
Cortical and Plain	15 (1.3%)	12 (3%)	
Plain	598 (53.6%)	211 (52%)	
Dihedral	82 (7.3%)	21 (5.1%)	
Facetted	140 (12.6%)	122 (30%)	
Crush and others	121 (10.8%)	28 (6.9%)	
Platform treatment	*n* = 1116	*n* = 406	Chi-square = 18.103, df = 1^a^, p<0.001
none	848 (76%)	263 (64.8%)	
Battering/chipping	267 (23.9%)	142 (35%)	
Grinding	1 (0.1%)	1 (0.2%)	
Platform thickness (mm)	*n* = 897	*n* = 336	t-value = 3.524, p<0.001
Min	0.3	1.27	
Max	37.4	19.7	
Mean	7.5	6.6	
SD	3.8	2.9	
Exterior platform angle (°)	*n* = 824	*n* = 323	t-value = -7.585, p<0.001
Min	45	57	
Max	113	105	
Mean	74	79	
SD	9.4	7.1	

^a^ only none and battering/chipping types are tested.

Previous studies of the 1980 collection indicated that direct percussion with a hard hammer was the main knapping technique used at SDG 1 [20], and our data are consistent with this. Pertinent observations include well demarcated percussion points on some specimens, frequent cortical and plain platforms, thick platforms, frequent and well-developed bulbs, and EPAs clustering around 75°-80° (Table 4). There is no clear difference in knapping technique for producing flakes and laminar blanks at SDG 1. Although the occasional use of soft stone hammer and marginal percussion has been suggested based upon the study of the 1980 collection [20], abrasion on the exterior edges of platforms and lipping of the internal edges are nearly absent in the 1963 collection. This is an important difference from the quasi-contemporaneous assemblages in northern Mongolia and the Transbaikal region, where soft hammer percussion was frequently used [18, 19].

### Retouched artifacts

**Fig 13:**
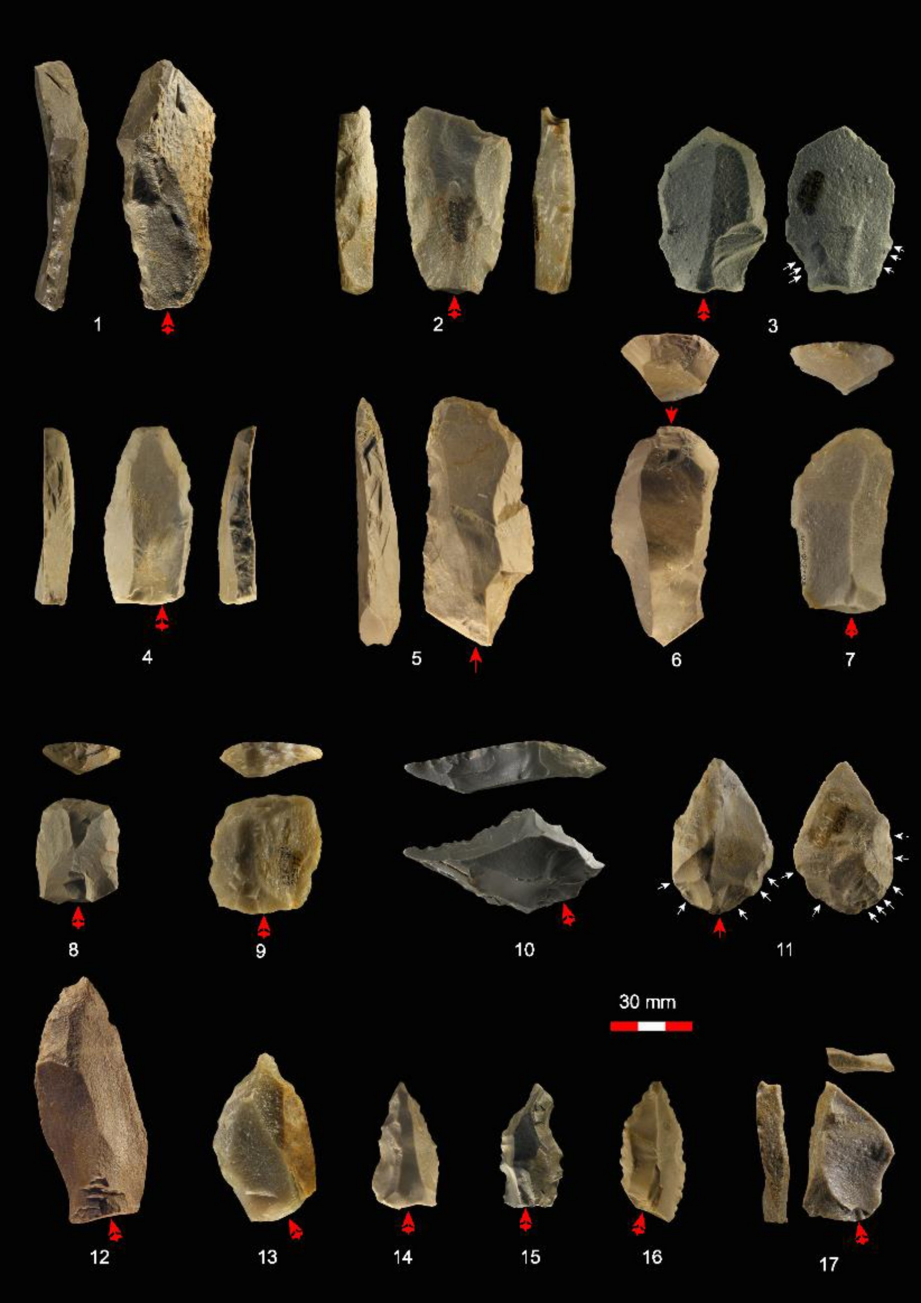
Examples of retouched pieces in the SDG 1963 assemblage from Layer 8: 1, single-edged scraper; 2, double-edged scraper; 3,4, convergent scrapers; 5–9, endscrapers; 10, denticulate; 11,12, points; 13, awl; 14–16, denticulated points; 17, burin.

A selected group of retouched artifacts from the 1963 assemblage from SDG 1 are illustrated in Fig 13. From a traditional typological point of view, retouched tool forms considered typical of both Middle and Upper Paleolithic tool-kits are common (Table 5). This is consistent with observations made by different scholars on the 1980 collection [20, 27]. The majority of the retouched edges were shaped unifacially: bifacial retouch is extremely rare. Two specimens show elaborate proximal modification with inverse (Fig 13: 3) and bifacial (Fig 13: 11) retouch, resulting in thinned bases probably for hafting. Simple (single-edged) sidescrapers (n = 94, 22.5%) make up the majority of the scraper group, following by convergent (n = 14), transverse (n = 12), double-edged (n = 9), and déjeté scrapers (n = 4). Blanks of simple scrapers are mainly flakes (67%). Denticulates (n = 130, 31.1%) and notched pieces (n = 30, 7.2%) are quite common and most of them are manufactured from flakes (73.8%). As for tool types more typical of UP assemblages, endscrapers (n = 48), burins (n = 7), and truncations (n = 21) make up to 18.2% of the complete tools. The UP tool forms were manufactured as frequently on blades (36.8%) as on flakes (38.2%). Pointed tools including awls (n = 13), points (n = 18), and denticulated points (n = 8) are abundant in the 1963 assemblage. A distinctive group of artifacts are eight points with convergent denticulated edges and acute tips: similar implements were also identified in the 1923 collection, and illustrated examples include figs 38 and 40 in [23]. Flakes were more often used as blanks for pointed tools (43.6%) than blades (30.8%). A few combination tools with more than one type of retouched edge have also been identified in the 1963 assemblage (n = 10, 2.4%).

**Table 5:** Tool types ^a^ in the SDG 1963 assemblage from Layer 8.

Typological categories	Numbers	On generalized flakes	On blades	On other blanks
Single-edged sidescraper	94	63	13	18
Double-edged sidescraper	9	6	3	0
Convergent scraper	14	11	2	0
*Déjeté* scraper	4	4	0	0
Transverse scraper	12	12	0	0
Denticulate	130	96	17	17
Notched piece	30	22	5	3
Borer/Awl	13	7	3	3
Combination tool	10	7	1	2
Denticulated point	8	5	2	1
Point	18	5	7	6
Truncation	21	10	9	2
Endscraper	48	27	14	7
Burin	7	1	5	1
**Total**	418	276 (66%)	81 (19.5%)	60 (14.5%)

^a^ tool fragments (n = 106) are not counted here.

Overall, flakes (66%) are more common than blades (19.5%) among the tool blanks, but for some types such as points and burins, blade blanks were more often used. On the other hand, the total number of complete flakes is much larger than the number of complete blades, and the fragmentation rate of blades is much higher than that of flakes (Table 4), so proportions of retouched pieces are different. In fact, knappers transformed a higher proportion of complete blades (38/181 = 21%) into various types of retouched tools than flakes (160/1117 = 14.3%).

Selection of blanks for retouch was further evaluated by the differences in overall morphology (length, width, and thickness) of blanks (Table 6). The mean length of flake tools is not significantly different from that of the unmodified flakes. On the other hand, the mean length of blade tools is significantly larger than that of blade blanks, despite the fact that many retouched blades (eg., endscrapers, points, truncations, and burins) would have been shortened by the modification of one end. This indicates that knappers at SDG 1 tended to select longer blades to retouch. Although retouched flakes and blades tend to be slightly wider and thicker than unretouched ones, the differences are not statistically significant for either type of blank.

**Table 6:** Selection of blanks for retouch in the SDG 1963 assemblage from Layer 8.

			Mean (mm)	Standard deviation	t-value	p
Length	flake	Unretouched (n = 937)	38.9	14.8	-1.316	.189
		Retouched (n = 179)	40.6	16		
	blade	Unretouched (n = 349)	43.2	13.6	-4.850	**<0.001**
		Retouched (n = 57)	56	19.2		
Width	flake	Unretouched	35.2	11.1	1.624	.105
		Retouched	33.6	13.5		
	blade	Unretouched	24.5	6.9	-2.982	.003
		Retouched	27.5	7.4		
Thickness	flake	Unretouched	10.8	4.5	-1.864	.063
		Retouched	11.5	4.5		
	blade	Unretouched	9.3	3.5	-2.296	.022
		Retouched	10.4	3.5		

## Discussion

### Intra-assemblage variation in the Paleolithic horizon at SDG 1

Previous studies of SDG1 assemblages, principally from the 1980 excavations, emphasized the mixture of features considered typical of Middle and Upper Paleolithic. More specifically, the core reduction sequences emphasize production of blades or laminar blanks from both hierarchical and volumetric exploitation of the raw material, and retouched tools include forms considered typical of both MP and UPs. This mixture of techno-typological attributes was observed in the 1963 collection as well. Because of uncertainties about the stratigraphy, it is possible that the “Paleolithic Layer 8,” which is at least two meters thick, actually contains a sequence of assemblages showing changing frequencies of MP and UP features. In other words, the particular mix of featured for which SDG 1 is known could in fact be the result of an *in situ* evolution from a basically Middle Paleolithic technology into a more classically Upper Paleolithic one.

However, an examination of the core reduction from nearby locality 9 does not support this scenario. At Shuidonggou locality 9, almost the entire assemblage comes from a single, 20-cm-thick layer beginning just below the ground surface. The relatively small number (n = 414) and limited distribution of artifacts suggest that this locality contains material from a single relatively brief occupation. Although the assemblage is small it is also quite diverse. Levallois-unidirectional/bidirectional and subprismatic blade cores were found at this locality [48]. The reduction sequences reconstructed closely resemble that of SDG 1. Unfortunately, retouched pieces are rare at locality 9. The core data at least suggest that the combination of techno-typological attributes seen at SDG 1 and other localities in the surrounding area is not a result of mixed assemblages but is in fact part of the definition of the IUP in northeast Asia. It is also important to emphasize that no assemblages with purely Middle Paleolithic features, or with Middle Paleolithic dates, have been found in the Shuidonggou area. Human occupation appears to have begun only around 45–40,000 years ago in the area.

On the other hand, it is possible that more than one period of occupation has been lumped together within the Paleolithic layer at SDG 1. At Shuidonggou locality 2, just across the river, core-flake assemblage appear to have replaced assemblages with large blades, occupying much of the Paleolithic sequence [31, 32]. It is entirely possible that one or more horizons with core-flake technologies is also represented in the thick Paleolithic deposits at SDG 1. Another possibility is the collection attributed to Layer 8 in fact contains some materials from Layer 7 which yielded mixed assemblages [24] due to the less accurate stratigraphic control of the 1963 excavation. The problem is that the simple cores and flakes found in most of the SDG 2 assemblages have few diagnostic characteristics. It would be difficult to distinguish the debitage of simple core-flake assemblages from that produced during initial core shaping and maintenance of prepared core reduction. In this respect it is important to recall that the 1963 assemblage contains 41 “simple cores” (18.8% of the complete cores), including tested pieces, chopper/chopping tools, simple unipolar, and polyhedral pieces. In the sample from the 1980 excavation there are 64 “simple cores” (38.8% of the complete cores) [27]. This differs strongly from other Initial Upper Paleolithic assemblages in northeast Asia, such as SDG 9 (n = 0) [48] and Kara Bom in the Siberian Altai (9.5% simple cores) [27]. Simple core forms could represent the initial stage of more elaborately prepared cores. If this were the case, simple core forms should be larger than more elaborately cores. However, the mean maximum size of simple cores in our assemblage (mean = 59.2 mm, sd = 21.1 mm) is essentially equal to that of prepared core forms (mean = 60.4 mm, sd = 16.4 mm; t-value = -0.409, p = 0.683). This raises possibility that the simple cores represent an independent reduction strategy at SDG 1, perhaps corresponding to one or more hitherto unrecognized later Paleolithic occupations.

Based on their study of the 1980 collection, Brantingham et al. [27] suggested that Shuidonggou locality 1 had a strong Middle Paleolithic typological signature. Indeed, so-called Middle Paleolithic tool types such as sidescrapers and denticulates are very frequent in the 1963 collection as well. Clearly, it is difficult to distinguish scrapers and denticulate tool made on flake blanks from a core-flake assemblage from similar tool forms made on flakes produced as byproducts of other reduction strategies. The higher proportion of simple core forms in the SDG 1 collections, and the frequency of simple core-flake assemblages post-dating the IUP at Shuidonggou, such as at localities 2, 7, and 8, suggest that a simple core-flake and scraper/denticulate-dominated assemblage could exist within the previous Paleolithic collections at SDG 1. Rather than representing a distinctive regional signature of the IUP, the strong “Middle Paleolithic” appearance of the SDG 1 retouched tool assemblage could thus be due to the mixing of different assemblages. The ongoing excavation at SDG1 with more accurate stratigraphic control should help to clarify this issue. As a consequence, in comparing the SDG 1 assemblage and other IUP assemblages in northeast Asia, it is best to focus on the laminar component due to the uncertain affiliation of other parts of the SDG 1 assemblage.

### Regional conservation and variations of the initial upper Paleolithic in northeast Asia

Although very broad comparisons among IUP assemblages have been made, technological details are still lacking for many assemblages. Here we choose sites from the Siberian Altai, northern Mongolia, and the Transbaikal region to examine some regional characteristics of IUP assemblages. We use Tostevin’s “behavioral domain” concept, simplifying it somewhat in order to apply it to a lower-resolution dataset available from the published literature on the IUP of northeast Asia. Tostevin [49–51] divided lithic operational sequences into five domains which represent independent behavioral decisions. These are “core modification,” “platform maintenance,” “direction of exploitation,” “blank production” and “tool manufacture.” These also constitute the main components of the middle part of a typical lithic *chaîne opératoire*. For our study, a check-list of various technological behaviors was applied to a variety of assemblages, with quantitative data included when possible. Table 7 shows that core reduction sequences are similar across IUP assemblages, particularly in the domains of core modification and the direction of the core exploitation. Initial flaking often began from the intersection of the broad and narrow faces of core blank, later expanding to the broad face during reduction. Removals from the broad core face were typically recurrent, without extensive repreparation between laminar removals. The initialization and maintenance of the core convexity was achieved by lateral crests or debordants, most often both. Bi-directional production of laminar blanks is common, and the abandoned cores are often flat. This indicates the general frameworks of blade production strategies are similar. Nevertheless, variation can be observed as well. In the domain of platform maintenance, platform preparation by faceting is common in most assemblages. On the other hand, significant inter-assemblage differences can be observed in other forms of platform modification. For instance, at the Kamenka site in the Transbaikal region, 29.9% of blade platforms were shaped by abrasion on the external edge. At Kamenka, platform abrasion is often associated with smaller platforms and a high frequency of ventral lips, features suggested to be consistent with a marginal percussion technique by soft-stone hammer [18]. High frequencies of blade platform abrasion are also observed at sites in northern Mongolia, such as Tolbor 16 [19]. However, this form of platform modification can be observed on fewer than 1% of blades at SDG 1. It is also rare in the Siberian Altai at localities such as Kara Bom [16].

**Table 7:** Laminar blank production behavior at Kara Bom, Tolbor 16, Kamenka, and SDG 1.

Technological Domains		Kara Bom [16, 48]	Tolbor 16 [19]	Kamenka [18]	SDG 1 (1963)
**Core modification**					
Core orientation	Recurrent use of longitudinal flat surface	√	√	N = 3, 30%	N = 94, 37.8%
	Recurrent use of longitudinal narrow surface	√	√	-	N = 48, 19.3%
	Burin core	√	N = 2	N = 3, 30%	N = 2, 0.8%
Core management	Debordant	√	√	N = 23, 15%	N = 248, 13.3%
	Lateral crest	√	√	√	N = 33, 1.8%
**Platform maintenance**					
Platform treatment	Facetted	√	23%	42.9%	N = 122, 30%
Platform modification	Chipping/pecking	√	12%	27.3%	N = 142, 35%
	Abrasion	Few	47%	29.9%	N = 1, 0.2%
EPA		-	-	-	Mean = 79°
Platform thickness		-	Mean = 5.1±3	Mean = 7.2±2.5	Mean = 6.6±2.9
**Direction of core exploitation**					
unidirectional		37% (OH5), 48% (OH6)	36%	N = 40, 25.6%	N = 48, 30.1%
bidirectional		52% (OH5), 43% (OH6)	30%	N = 68, 43.6%	N = 55, 35.3%
**Dorsal surface convexity**					
length/width ratio		-	-	Mean = 2.4	Mean = 2.3
width/thickness ratio		-	-	Mean = 3.2	Mean = 2.6

Quantitative comparison of retouched tool assemblages among different IUP assemblages in northeast Asia is difficult at present because of limited data. Upper Paleolithic tool types including endscrapers, burins, and truncations appear in varying frequencies among assemblages, alongside sidescrapers, a category into which we also place retouched blades. For instance, UP formal tools make up 32–39% of the retouched tools from IUP assemblages at Kara Bom [52]. This is higher than in the SDG 1 assemblage overall (17.8%), but considering only laminar blanks, as suggested above, UP formal tools make up 34.6% of retouched pieces at SDG 1. The frequency of UP formal tools is much lower at other northeast Asian IUP sites such as Kemenka (8.6%) and Tolbor 16 (0.4%) [18, 19]. Pointed tools are present in many sites but the denticulated points at SDG 1 appear to be unique based on the published literature.

The comparison of the IUP assemblages from the Siberian Altai, northern Mongolia, the Transbaikal region, and northern China demonstrates that the IUP in northeast Asia is technologically coherent and conservative. This is not very surprising, as the IUP is defined based largely on technological features. The overall similarities in blank production are consistent with a strong diffusion model [49, 51] for the northeast Asian IUP. In light of the distribution of radiocarbon dates, it seems that the IUP of northern Mongolia, the Transbaikal region, and northern China dispersed from the Siberian Altai [53, 54]. The use of marginal soft-hammer percussion, and attendant modification of platform edges appear most frequently in the northern Mongolia and Transbaikal regions, but not other regions. This could imply that this knapping technique developed as a regional adaptation after IUP arrived in this area. One possible factor responsible for this could be the raw materials used in different regions, although this should be investigated thoroughly in another context. Scholars have proposed that the IUP at SDG 1 dispersed from Mongolia, based on the later age of SDG 1 [27, 55]. However, more recent findings suggest that the IUP of SDG is earlier than previously thought [31, 33, 34]. Combined with the uneven distribution of the marginal percussion technique, it is more like the IUP in northern China and the northern Mongolia-Transbaikal regions were associated with independent dispersals from the Siberian Altai. The distinctive denticulated retouched points at SDG 1 may also be a regionally derived development. However, the mechanisms behind these regional signals should be explored integrating data from different regions.

Another distinctive *negative* feature of the IUP in the SDG area is the lack of ornaments. A large variety of personal ornaments have been reported in association with IUP assemblages in the Altai, Mongolian, and Transbaikal regions, although the total number of such objects is actually quite limited [52, 56]. Ornaments reported include ostrich eggshell beads, perforated teeth and shells, incised bone and ivory pendants, etc. A single ostrich eggshell bead from the 1963 excavation campaign at SDG 1 was mentioned in a review paper [35], but the context is not certain. It is possible the lack of ornaments at SDG 1 results from the coarse excavation and recovery practices in the earlier excavations. Whether the scarcity of ornaments in the IUP horizon is a regional feature of the Shuidonggou area or is a product of the quality of recovery in old excavations can only be confirmed by the ongoing excavation. It is worth noting that other forms of symbolic behavior seem to have been practiced by the Paleolithic inhabitants of SDG 1: a siliceous dolomite core with engraved lines was reported from the 1980 collection [57].

## Conclusion

In this paper we have presented results from a techno-typological study of the lithic artifacts from the 1963 excavations at SDG 1, an assemblage which has not been examined previously in detail. Although the stratigraphic context of Paleolithic deposit of SDG 1 is somewhat uncertain, the lithic assemblage is relatively intact which helps to provide a full picture of lithic technology at the site. Two broadly-defined classes of core reduction strategy can be identified at SDG 1, including simple, unsystematic core reduction and prepared core reduction. The mean size of simple cores is not significantly larger than that of prepared cores which indicates simple core reduction may not be the initial stage of the prepared core reduction at SDG 1. Instead, it is more likely an independent reduction strategy. The simple core reduction component may due to mixing with a simple core-flake assemblages from different archaeological layers, especially layer 7. Among the prepared core reduction sequences, the main reduction strategy shows an asymmetrical method producing blades and elongate flakes from one broad face of the core. This method resembles a recurrent Levallois blade method *sensu lato*. Other strategies involving prepared cores resulted in edge-faceted cores, prismatic/subprismatic cores, and burin cores, although the number of the last category is small. Typologically, retouched tools show a mix feature of typical MP and UP tool types. The higher proportion of typical MP tool types may also be partially a product of mixed assemblages.

Because of the possibility of mixing, only artifacts made on the products of blade production systems were used in comparisons with assemblages from other regions. The comparison of IUP assemblages from different parts of northeast Asia show that IUP assemblages form a coherent technological complex, but some regional variations are also present. The assemblage from SDG 1 is more similar to material from the Siberian Altai than to material from northern Mongolia and the Transbaikal region, despite the fact that the later are geographically closer. This suggests that the SDG IUP technology may have dispersed from the Siberian Altai independently instead of from northern Mongolia as others have proposed [11]. We argue that a multi-directional model of diffusion of the IUP in northeast Asia would be more appropriate than a unilineal one. Due to uneven availability of data from different regions there is ample room for refining or replacing the model in the future. These observations also point to larger questions. The most obvious questions center on how local variants of the IUP developed, and the roles that raw materials, functional or economic constraints, and patterns of social transmission played in enhancing or constraining diversity. A broader question concerns how hominins producing the IUP maintained continuity in technology over such a vast area in northeast Asia. Although the existing collections from older excavations are plagued by uncertainties relating the stratigraphic context and chronology, recent excavations with better contextual control in northern Mongolia such as Tobor 16, reinvestigation of the Kara Bom site in the Siberian Altai, and the most recent program of excavation at SDG 1 will go a long way toward clarifying the genesis and significance of the IUP phenomenon in eastern Eurasia.

## Supporting information

S1 Data (XLSX)
